# The associations of COVID-19 percent positivity rate, relationship quality, and season with daily anxiety and depression in couples living in NYC

**DOI:** 10.3389/fpsyg.2022.968243

**Published:** 2022-12-22

**Authors:** Talea Cornelius, Ana M. DiGiovanni, Niall Bolger

**Affiliations:** ^1^Center for Behavioral Cardiovascular Health, Columbia University Irving Medical Center, New York, NY, United States; ^2^Department of Psychology, Columbia University, New York, NY, United States

**Keywords:** COVID-19, couples, dyads, relationship quality, anxiety, depression, NYC

## Abstract

**Background:**

The COVID-19 pandemic changed nearly every aspect of daily life and had detrimental effects on mental health. Yet, impacts have been heterogeneous. We tested whether fluctuations in local COVID-19 percent positivity rates were associated with daily anxiety and depression in couples living in NYC, as well as whether these associations varied by relationship quality or season. We expected that adverse impacts of COVID-19 may be attenuated by high-quality relationships and during warmer months, or that people may habituate over time.

**Methods:**

Data on seven-day rolling average COVID-19 percent positive rate each day in NYC were merged with a 14-day dyadic diary study of cohabiting couples living in NYC between August 2020 through April 2021 (232 individuals from 116 couples; mean age 28.42 years, 52.59% female, 53.02% White). Dyadic multilevel models estimated the association COVID-19 positivity rate, season (sine and cosine of the calendar date), baseline relationship quality, and all two-and three-way interactions of these variables with daily anxiety and depression. Covariates included weekend and COVID-positive case within the couple.

**Results:**

Anxiety and depression mirrored COVID-19 positivity rates, and there was some evidence for habituation over time. Significant two-and three-way interactions suggested that being in a high-quality relationship buffered the association of COVID-19 positivity rate with both anxiety and depression during months when cases were low. Anxiety was elevated for individuals in high- (v. low-) quality relationships during the December–January surge.

**Conclusion:**

Seven-day rolling average COVID-19 percent positivity rate was associated with daily anxiety and depression among couples living in NYC. There was some evidence that individuals habituated to this stressor over time and that high-quality relationships were protective for mental well-being; however, there was some suggestion that couples in high-quality relationships may have engaged in processes such as co-rumination during surges, worsening their daily anxiety.

## Introduction

The stress associated with the COVID-19 pandemic, including surges in infection rates and the implementation of stay-home and social-distancing policies, has had serious mental health consequences for individuals. Indeed, numerous studies have documented a high prevalence of anxiety and depression in the general population during the pandemic (Choi et al., 2020; Ettman et al., 2020; McGinty et al., 2020; Salari et al., 2020; Twenge and Joiner, 2020; Kwong et al., 2021; Nochaiwong et al., 2021; Santabárbara et al., 2021). However, COVID-related stressors have not remained stagnant, as the context of stress exposure has varied considerably based on living situation and external factors such as temperature. First, positivity rates have changed considerably over time – both increasing and decreasing (Hong et al., 2021; COVID-19, 2022) – suggesting variable exposure to the stress of COVID-19 over the past two plus years. In terms of context, some months allow for the maintenance of safer, socially distanced activities (e.g., outdoor dining with friends during warmer months), removing some of the stress associated with isolation and monotonous routine. People cohabiting with a significant other may also be protected against the environmental stress exposure of surges in COVID-19 infections, particularly those individuals in romantic relationships that are high-quality (Lillie et al., 2021; Randall et al., 2021). Finally, as time has passed, people may also habituate and become accustomed to COVID-19 as a “new normal.” Most studies of mental health during the pandemic have not directly examined the association of fluctuations in COVID-19 infection rates with daily reports of anxiety and depression; research has also not examined how relationship quality may moderate these effects. In this study, we test whether environmental exposure to greater COVID-19 stress, as measured by the rolling average percent positive testing rate over the past 7 days, is associated with worse anxiety and depression each day in cohabiting couples living in NYC – an early epicenter of the COVID-19 pandemic (Dong et al., 2020). We additionally test whether these associations exhibit seasonal patterns, given temperature variability across seasons, and test whether relationship quality buffers the adverse impact of COVID-19 infection rates on mental health.

### COVID-19 and mental health

COVID-19 is a global stressor that has profoundly changed the social landscape. It has also had significant impacts on mental health, with recent work reporting a three-fold increase in anxiety (Santabárbara et al., 2021) and depression (Ettman et al., 2020) in the general population, and another study finding a three-fold increase in positive screens for mood disorders, including anxiety and depression, between early 2019 and early 2020 (Twenge and Joiner, 2020). Despite universal exposure to this environmental stressor, some individuals have exhibited resilience and adaptation. Specifically, research by Sauer et al. (2020) found that, although higher levels of health anxiety during the pandemic were associated with a significant initial elevation in COVID-related anxiety, those with higher health anxiety also showed severe dampening in their COVID-related health concerns over time (Sauer et al., 2020). This suggests that the negative effects of the pandemic do not indefinitely increase during the pandemic and that people habituate to the reality of COVID-19 even as it continues to impact the world. Some research also suggests that those individuals who were able to maintain stable in-person social contact did not experience adverse mental health consequences. In a sample of college students living in the Netherlands, Fried and colleagues (2021) found decreases in anxiety, loneliness, and COVID-19 concerns over a two-week span (Fried et al., 2020). Thus, the ability to engage in normal social activities, for example, during warmer months, may protect against adverse mental health consequences.

Despite the changing nature of the COVID-19 pandemic, essentially no research has directly examined how these contextual factors (i.e., changes in environmental stress exposure, temperature, time) impact psychological well-being. Informed by Bronfenbrenner’s Ecological Systems Theory, we conceptualize COVID-19 positivity rates as an essential part of an individuals’ surrounding exosystem—that is, the indirect environmental circumstances that impact individuals (e.g., their neighborhood, healthcare policies, governing agencies; Bronfenbrenner, 1992). Importantly, these environmental factors do not have to operate within conscious awareness in order to have a significant impact on individuals, health and well-being (Cook et al., 2014). This macro-level conceptualization of a major stressor is thus an inescapable lived reality, and understanding its impacts has important implications for public health. It is also critical to account for the fact that different environmental components interact with each other to determine overall impact. For example, as COVID-19 wore on, policies were put in place to enable continuation of “normal” activities (e.g., allowing takeaway cocktails, opening new outdoor dining spaces). Similarly, it was easier to maintain social activities outdoors during warmer months, minimizing risk exposure. A complete picture of this environmental system must consider these factors in addition to mere COVID-19 positivity rates themselves.

### Relationship quality as a buffer

Another central system that shapes individual’s health and well-being is the mesosystem (Bronfenbrenner, 1992), which includes closer relationships. Because surges in COVID-19 infection rates have been accompanied by potentially isolating policies, particularly early during the pandemic, it is particularly important to consider couple-level factors that may alter the association of COVID-19 infection rates with mental health. Indeed, early COVID-19-related business closures, social distancing policies, quarantine due to COVID-19 illness, and other factors, may have left many individuals with access only to those in their own home. This makes cohabitating romantic partnerships a central context for determining the impact of the COVID-19 pandemic on anxiety and depression. Although research has examined how COVID-19 stressors impact relationship quality (Balzarini et al., 2020; Williamson, 2020; Neff et al., 2021), few have considered how relationship quality may buffer the negative impacts of COVID-19 on individual-level outcomes such as anxiety and depression (Cornelius et al., 2021). Drawing from theories that emphasize the ways in which supportive relationships can mitigate harmful effects of stress exposures (Pearlin et al., 1981; Cohen and Wills, 1985; Taylor, 2011), it follows that individuals who report higher levels of relationship quality might be better equipped to deal with a stressful exosystem.

### The current study

The current study is among the first to situate individual well-being within both environmental and relational systems and to examine the interactions between them. We extend prior research examining the impact of COVID-19 on mental health in a general population by testing the hypothesis that greater exposure to COVID-19, as measured by seven-day rolling average daily percent positive testing rate, would be associated with worse anxiety and depression each day. We additionally investigated whether this association would vary by season and over time, given that people may have become more accustomed to the realities of the COVID-19 pandemic as it became “the new normal,” in addition to the fact that it was easier to maintain social activities in a socially distanced manner and less risky manner during warmer months. Finally, we tested whether higher quality relationships, as measured at baseline, are associated with better mental health outcomes and, importantly, whether baseline reports of relationship quality buffer the adverse impact of fluctuating COVID-19 positivity rates on mental health. Although we had expectations about the direction of these moderation effects, we considered these analyses to be exploratory and descriptive and focused on plotting results to aid interpretation; we therefore did not pre-register any hypotheses. To accomplish this, we combined publicly available data from NYC on COVID-19 percent positivity with data from a 14-day dyadic daily diary of cohabiting couples living in NYC between August 2020 through April 2021. All data and code for analyses can be found on the Open Science Framework.^1^

## Materials and methods

### Procedure

This study comprised a baseline survey followed by 14 days of daily diary data collected between August 2020 and April 2021. Recruitment efforts included online postings (e.g., Facebook, Craigslist, Twitter, Honeybee Hub, University Listserv), word of mouth, and flyers in NYC, requesting couples to participate in a 14-day diary study about the social and psychological impacts of the COVID-19 pandemic. Couples were screened for eligibility via a brief Qualtrics screening survey. If eligible, research assistants emailed these couples to schedule a Zoom call where eligibility was confirmed and additional study information was provided. Next, research assistants emailed each member of the couple separately to provide electronic informed consent and complete a baseline survey. If both members of the couple consented and completed the baseline assessment, the couple then entered the daily diary portion of the study.

Diary cohorts always began on a Tuesday evening and diaries were sent to participants at approximately 7 pm each night. Participants were instructed to fill these diaries out without their partner present around the time they were going to sleep. If participants completed the baseline survey and at least 80% of the daily diaries (11 out of 14) on the correct day, they were paid either $20, $30, or $40 (payment was increased over the course of the study to bolster recruitment). Diary entries completed outside the hours of 6 pm – 3 am were excluded, as were entries where the participant failed one or more attention checks or responded in under 2 mins (49 diary entries total). All procedures were approved by the Columbia University Institutional Review Board and all participants provided electronic informed consent prior to beginning the baseline assessment.

Note that couples were enrolled in the study for a 14-day period only; thus, the sample of couples varied over the course of the observation period. Supplemental analyses (not shown) found no significant evidence that date of enrollment was associated with couples’ age or relationship quality.

### Participants

Eligibility criteria for the larger study included age 21 or older and currently cohabitating with a romantic partner in either NYC, Hoboken, Newark, or Jersey City. Exclusion criteria included living with anyone besides their romantic partner, such as a roommate or children. After excluding individuals who did not fit our eligibility criteria (for more details on excluded observations, see DiGiovanni et al., 2022), we had a total of 126 couples (252 individuals) who completed baseline surveys and were sent daily diaries; 120 of these couples had data where both members of the dyad completed one or more diary entry. For the present analysis, we selected only those couples with a NYC zip code (118 couples) and excluded those with missing data, leaving us with a final sample size of 116 couples. Sample size was based solely on feasibility; a power analysis was not conducted. The average number of daily diaries completed by participants in the dataset were 9.85 diary days out of 14 total possible days (*Median* = 11 days).

### Measures

#### COVID-19 positivity rate

Data on citywide COVID-19 percent positive testing rate, calculated as seven-day rolling average for the number of positive tests divided by total number of tests each day were downloaded from the NYC Department of Health and Mental Hygiene github repository (Coronavirus Data [Internet], 2022). A rolling average was used to smooth over delays in daily reporting. These data were merged with the daily diary data by date.

#### Seasonal variation

Seasonal variation was modeled as sine and cosine of the date of each daily diary report, spanning the data collection period which ran from August 2020 through April 2021 (Ma et al., 2006; Coronavirus Data [Internet], 2022). Time was additionally modeled using linear and squared terms in supplementary analyses to represent linear time counting up from the study start date, instead of sine and cosine of the calendar date, which assesses seasonal variation.

#### Relationship quality

Relationship quality was measured at baseline using the Quality of Marriage Index (Norton, 1983). Items were adapted to talk about “your relationship with your partner.” Five items are scored from 1, “Strongly disagree,” to 7, “Strongly agree,” and ask questions such as “We have a good relationship,” and “My relationship with my partner makes me happy.” A sixth item asks about the degree of happiness in the relationship and ranges from 1, “Extremely low,” to 10, “Extremely high.” Items are summed, such that higher total scores indicate higher relationship quality (*Cronbach’s α* = 0.94).

#### Anxiety and depression

Primary outcomes are daily reports of anxiety and depression, assessed using an emotion rating battery adapted from the Profile of Mood States (Shacham, 1983) from a daily diary study which assessed mood and stress in couples where one partner was approaching the NY State Bar Exam (Shrout et al., 2006). Each dyad member independently rated the extent to which they were experiencing certain emotions each day using the question stem, “Please rate the extent to which you are feeling or experiencing these feelings or emotions RIGHT NOW, IN THE EVENING.” Response options ranged from 1, “not at all,” to 9, “extremely.” Daily anxiety was calculated by taking the average of three items: “on edge,” “uneasy,” and “anxious.” Daily depression included four items: “discouraged,” “blue,” “hopeless,” and “sad.”

#### Covariates

Time-varying covariates included whether or not it was a weekend and whether or not there was a suspected or confirmed COVID-19-positive case in the couple, with each variable dummy-coded such that 1 indicated, “yes,” and 0 indicated, “no.”

#### Demographics

Demographics were self-reported at baseline and included age, sex, gender, race, ethnicity, relationship length, residence type, work status, and current income.

All other variables that were included in the baseline and daily diary can be found on the Open Science Framework.^2^

### Data analysis strategy

Dyadic mixed multilevel models for indistinguishable dyads were estimated (Kenny et al., 2006; Kashy et al., 2008) to examine two daily outcomes for each dyad member: daily anxiety and daily depression. Models included a random intercept for each dyad member, a covariance between dyad-members’ intercepts, a random slope for the association of citywide percent positive rate with daily anxiety and depression for each dyad member, a covariance between dyad-members’ slopes, an intercept-slope covariance assumed to be the same for each dyad member (both within and across individuals), and a covariance between the daily anxiety/depression residuals of dyad members. The primary predictors were seven-day rolling average citywide COVID-19 percent positivity testing rate, date (modeled as sine and cosine of the date), and baseline relationship quality. Effect modification was tested by including the multiplicative interaction of COVID-19 percent positivity rate, season, and relationship quality. Models were estimated first with main effects only, then two-way interaction terms were tested, and finally three-way interaction terms were tested. All models controlled for weekend and whether there was a suspected or confirmed COVID-19-positive case within the couple, and simple main effects for relationship quality were examined for the 5^th^ of each month covered during the data collection period.

Example SAS syntax for the main effects model predicting anxiety is below:

**proc mixed** data = osf_data noclprint method=ml covtest;

class dyadid day_a;

model anx_a = weekend covid_couple sinx cosx

qmi_c_a citywide

/ covb s cl;

random pers_one pers_two pers_one*citywide

pers_two*citywide /

subject=dyadid type=lin**(6)** ldata = CovMatrix gcorr;

repeated / type=cs subject=dyadid*day_a;

**run;**

**Figure 1:**
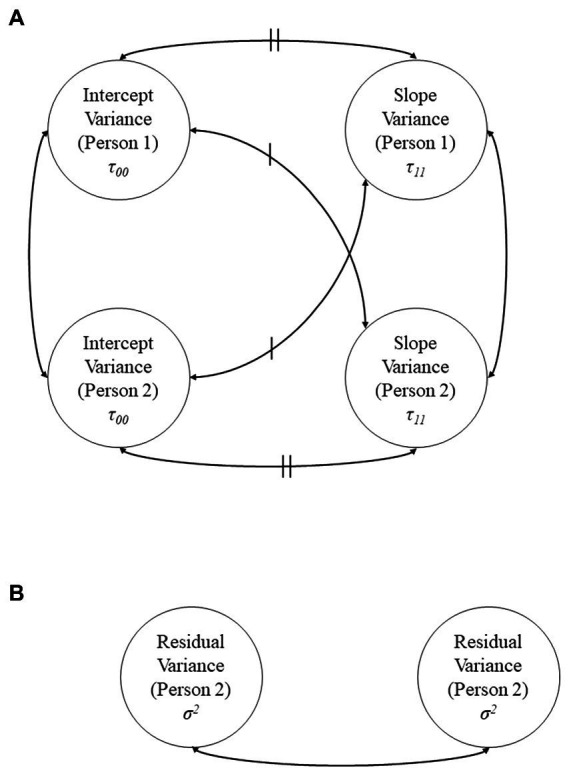
Conceptual diagram showing all modeled random effects and their covariances (**A**) and residuals and the within-dyad residual covariance (**B**). Intercept and slope variances are restricted equal within dyads (i.e., *τ*_00_ = *τ*_00_ and *τ*_11_ = *τ*_11_), as are residuals (i.e., *σ^2^* = *σ^2^*). Curved double-headed arrows represent covariances, and hashmarks denote covariances that have been restricted equal.

*anx_a* is daily anxiety, *weekend* is whether or not it was a weekend day (yes/no), *covid_couple* is whether or not the couple had suspected/confirmed COVID-19 (yes/no), *sinx* and *cosx* are sine and cosine of the date, respectively, *qmi_c_a* is relationship quality, and *citywide* is citywide COVID-19 percent positivity rate. The random statement includes intercepts for each dyad member and a random slope for the association of COVID-19 percent positivity with anxiety and depression for each dyad member; these variances and covariances have been constrained equal using a linear constraint matrix [*lin*(*6*)] because the dyads are indistinguishable, as they could not be distinguished based on gender, sex, or any other meaningful variables. A repeated statement was included to allow for correlated residuals within dyads each day. A conceptual diagram depicting these random effects, residuals, and their covariances is shown in Figure 1.

In addition to primary analyses, four sets of sensitivity analyses were conducted. In the first, we replaced the terms for sine and cosine of the date with linear and squared terms for continuous time (counting up from the study start date). In the second, we modeled linear and squared terms for time and additionally included season as a categorical covariate. In the third, we adjusted for COVID-19 vaccination status. Data on vaccination status were not collected until the very end of the study, as vaccines were not available at the beginning of the COVID-19 pandemic. We assumed that those who were missing values were not vaccinated. Finally, we adjusted for participant age, relationship length, race (White v. other), ethnicity, and gender (man v. other).

## Results

Of the 118 couples who provided an NYC zip code and were matched to COVID-19 percent positivity data, two couples were excluded due to missing data (e.g., data on anxiety or depression). This resulted in a final sample of 232 individuals from 116 couples. Mean age was 28.42 (*SD* = 7.26), 110 participants were assigned male at birth (47.41%), and 122 were assigned female at birth (52.59%). Most couples were opposite-sex (*n* = 100, 86.21%), with 16 same-sex couples (13.79%), and mean relationship length was 4.42 years (*SD* = 3.93). Just over half identified as a woman (*n* = 118, 50.86%), 109 identified as a man (46.98%), and five reported being genderfluid/genderqueer (2.16%). Most participants were White (*n* = 123, 53.02%), followed by Asian (*n* = 55, 23.71%), other (*n* = 21, 9.05%), more than one race (*n* = 18, 7.76%), Black (*n* = 13, 5.60%), and American Indian (*n* = 2, 0.86%). Thirty-five reported Hispanic ethnicity (15.09%). Most (*n* = 216, 93.10%) lived in an apartment, 69 were working in-person (29.74%), 60 were working from home (25.86%), and 103 were not working (44.40%). Of the 181 reporting a non-zero income, median current income was $57,000 (*IQR* = $58,000).

Averaged across the diary period, mean anxiety was 2.73 (*SD* = 1.42) and depression was 2.23 (*SD* = 1.22). Mean COVID-19 citywide percent positive rate was 4.09 (*SD* = 2.58; *Range* 0.90–8.76). Participants completed a median of 11 diary days (out of 14; *IQR* 8–12).

### Anxiety

**Figure 2:**
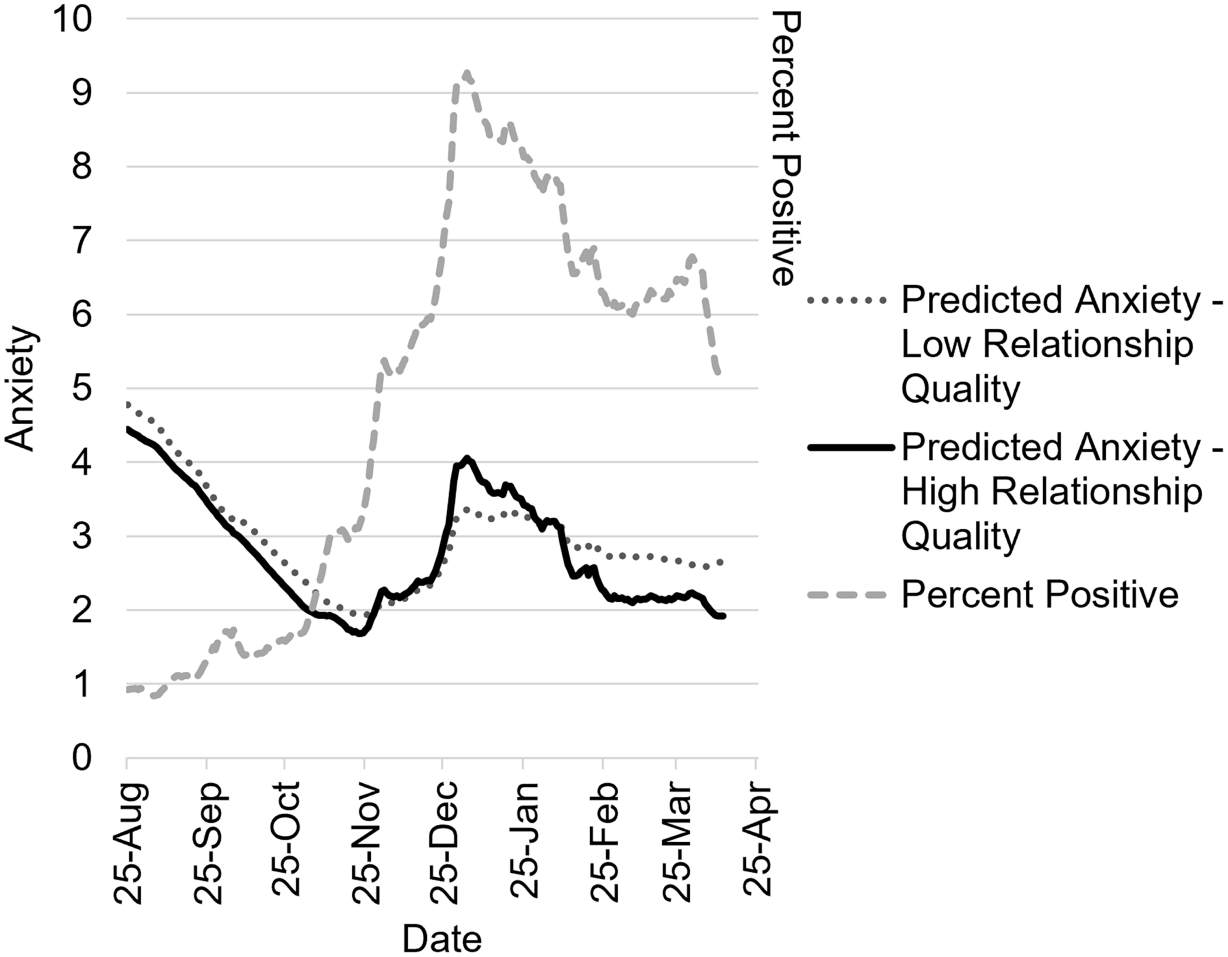
Two-way interaction model predicting daily anxiety between August 2020–April 2021 as a function of COVID-19 percent positivity testing rate, season, and relationship quality. Predicted daily anxiety in individuals with low-quality (−1 *SD*) and high-quality (45, maximum score, since +1 *SD* was slightly outside the range of the scale) relationships as a function of daily percent positive testing rate in NYC and season. Anxiety scores can range from 1 to 9, and percent positive can range from 0 to 100, but the range of percent positive was 0.84 to 9.14 during the observation period. The jagged shape of the graph comes from modeling the effect of actual reported citywide seven-day percent positivity rate.

**Figure 3:**
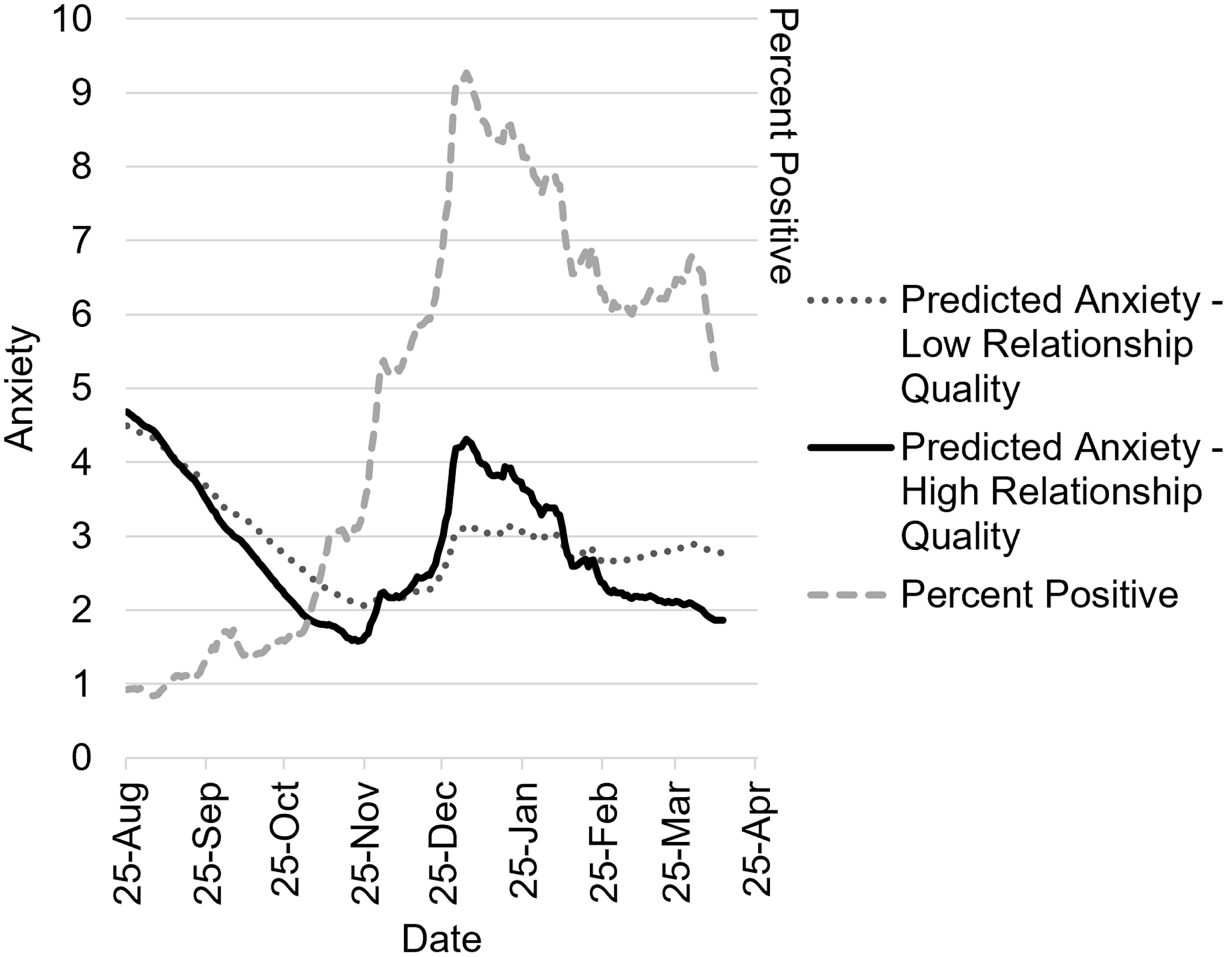
Three-way interaction model predicting daily anxiety between August 2020–April 2021 as a function of COVID-19 percent positivity testing rate, season, and relationship quality. Predicted daily anxiety in individuals with low-quality (−1 *SD*) and high-quality (45; maximum score, since +1 *SD* was slightly outside the range of the scale) relationships as a function of daily percent positive testing rate in NYC and season. Anxiety scores can range from 1 to 9, and percent positive can range from 0 to 100, but the range of percent positive was 0.84 to 9.14 during the observation period. The jagged shape of the graph comes from modeling the effect of actual reported citywide seven-day percent positivity rate.

Full model results for fixed effects are in Table 1. In the main effects model, days with a higher COVID-19 positivity rate were associated with greater daily anxiety, *B* = 0.23, 95% CI 0.03, 0.43, *p* = 0.023, and higher relationship quality was associated with lower anxiety, *B* = −0.04, 95% CI −0.07, −0.01, *p* = 0.014. Sine and cosine were significant at *p* < 0.001, suggesting seasonal variation in daily anxiety. In the two-way interaction model, the interaction of percent positivity with relationship quality was marginally significant, *p* = 0.07, as was the interaction of cosine and relationship quality, *p* = 0.054. This suggested that the association of baseline relationship quality with daily anxiety varied according to daily COVID-19 percent positivity rate and season. Sine and cosine interacted significantly with citywide positivity rate, *p* < 0.001, and, *p* = 0.002, respectively, suggesting that the association of daily COVID-19 percent positivity rate with daily anxiety varied by season. These interactions are depicted in Figure 2. The final model included a marginally significant three-way interaction of sine, relationship quality, and citywide positivity rate, *p* = 0.074; this is depicted in Figure 3 to aid interpretation. In both figures, daily anxiety is elevated during August 2020, but falls steadily over subsequent months, through November, with those in high-quality relationships exhibiting a slightly steeper decline. When cases spike in December, anxiety also spikes; counter to hypotheses, those in high-quality relationships exhibit a greater increase in anxiety as compared to those in lower quality relationships. As COVID positivity declines again through January – March, anxiety also decreases, and this is more apparent for individuals in high-quality relationships.

**Table 1:** Estimates for main effects model (column 1), two-way interactions (column 2), and three-way interactions (column 3) predicting daily anxiety.

	*B* [95% CI]	*B* [95% CI]	*B* [95% CI]
*Intercept*	3.60 [2.17, 5.03]**	7.54 [3.77, 11.31]**	4.81 [−0.09, 9.71]+
*Weekend*	−0.40 [−0.52, −0.28]**	−0.38 [−0.51, −0.26]**	−0.38 [−0.50, −0.26]**
*COVID Positive*	−0.11 [−0.5, 0.28]	0.01 [−0.38, 0.40]	0.03 [−0.36, 0.41]
*Sine*	1.05 [0.47, 1.63]**	2.13 [−1.15, 5.41]	−1.40 [−6.68, 3.89]
*Cosine*	−1.14 [−1.75, −0.53]**	1.24 [−1.79, 4.27]	0.50 [−3.82, 4.82]
*Relationship Quality (RQ)*	−0.04 [−0.07, −0.01]*	−0.12 [−0.21, −0.03]**	−0.05 [−0.17, 007]
*Percent Positivity Rate (PPR)*	0.23 [0.03, 0.43]*	−0.97 [−2.00, 0.06]+	−0.06 [−1.53, 1.41]
RQ*PPR		0.02 [−0.00, 0.05]+	0.00 [−0.04, 0.04]
Sine*RQ		0.00 [−0.07, 0.08]	0.09 [−0.04, 0.22]
Cosine*RQ		−0.07 [−0.14, 0.00]+	−0.05 [−0.16, 0.05]
Sine*PPR		−0.41 [−0.65, −0.18]**	0.69 [−0.54, 1.92]
Cosine*PPR		0.29 [0.11, 0.48]**	−0.01 [−1.16, 1.13]
Sine*RQ*PPR			−0.03 [−0.06, 0.00]+
Cosine*RQ*PPR			0.01 [−0.02, 0.04]
	*Est (se)*	*Est (se)*	*Est (se)*
*Intercept Variance (Common to Each Dyad Member)*	3.59 (0.60)**	4.11 (0.71)**	4.05 (0.70)**
*Between Dyad Member Intercept Covariance*	1.41 (0.60)*	1.91 (0.71)**	1.85 (0.70)**
*Slope Variance (Common to Each Dyad Member)*	0.19 (0.05)**	0.22 (0.06)**	0.22 (0.06)**
*Between Dyad Member Slope Covariance*	0.09 (0.05)*	0.12 (0.06)*	0.12 (0.06)*
*Within Dyad Member Intercept-Slope Covariance*	−0.74 (0.18)**	−0.88 (0.22)**	−0.88 (0.22)**
*Between Dyad Member Intercept-Slope Covariance*	−0.35 (0.17)*	−0.47 (0.21)*	−0.47 (0.21)*
*Daily Residual Variance (Common to Each Dyad Member)*	1.07 (0.05)**	1.07 (0.05)**	1.07 (0.05)**
*Between Dyad Member Daily Residual Covariance*	0.34 (0.05)**	0.32 (0.05)**	0.32 (0.05)**

+*p* < 0.10, *p < 0.05, **p < 0.01.

To probe the two-and three-way interactions, we estimated the association of relationship quality with daily anxiety on the 5^th^ of each month during data collection. Full results can be found in Supplementary Table 1. In the two-way interaction model, the association of relationship quality with lower anxiety was negative and significant on March 5^th^, *B* = −0.05, 95% CI −0.10, −0.01, *p* = 0.02, such that higher relationship quality was associated with decreased anxiety on March 5^th^. No other dates included in the simple slopes analyses (i.e., the 5^th^ of each month) were significant.

In the three-way interaction model, the association of relationship quality with greater anxiety was positive and marginally significant on January 5^th^, *B* = 0.10, 95% CI −0.01, 0.21, *p* = 0.073, such that higher relationship quality was associated with greater anxiety on January 5^th^. On November 5^th^, the association of relationship quality with anxiety was negative and marginally significant, *p* = 0.077. This marginally significant negative association emerged again by March 5^th^, *p* = 0.094, and relationship quality was associated with significantly lower anxiety on April 5^th^, *B* = −0.08, 95% CI −0.15, −0.01, *p* = 0.019. In other words, on the 5^th^ of November, March, and April, couples with higher (v. lower) relationship quality experienced decreased anxiety.

### Depression

**Figure 4:**
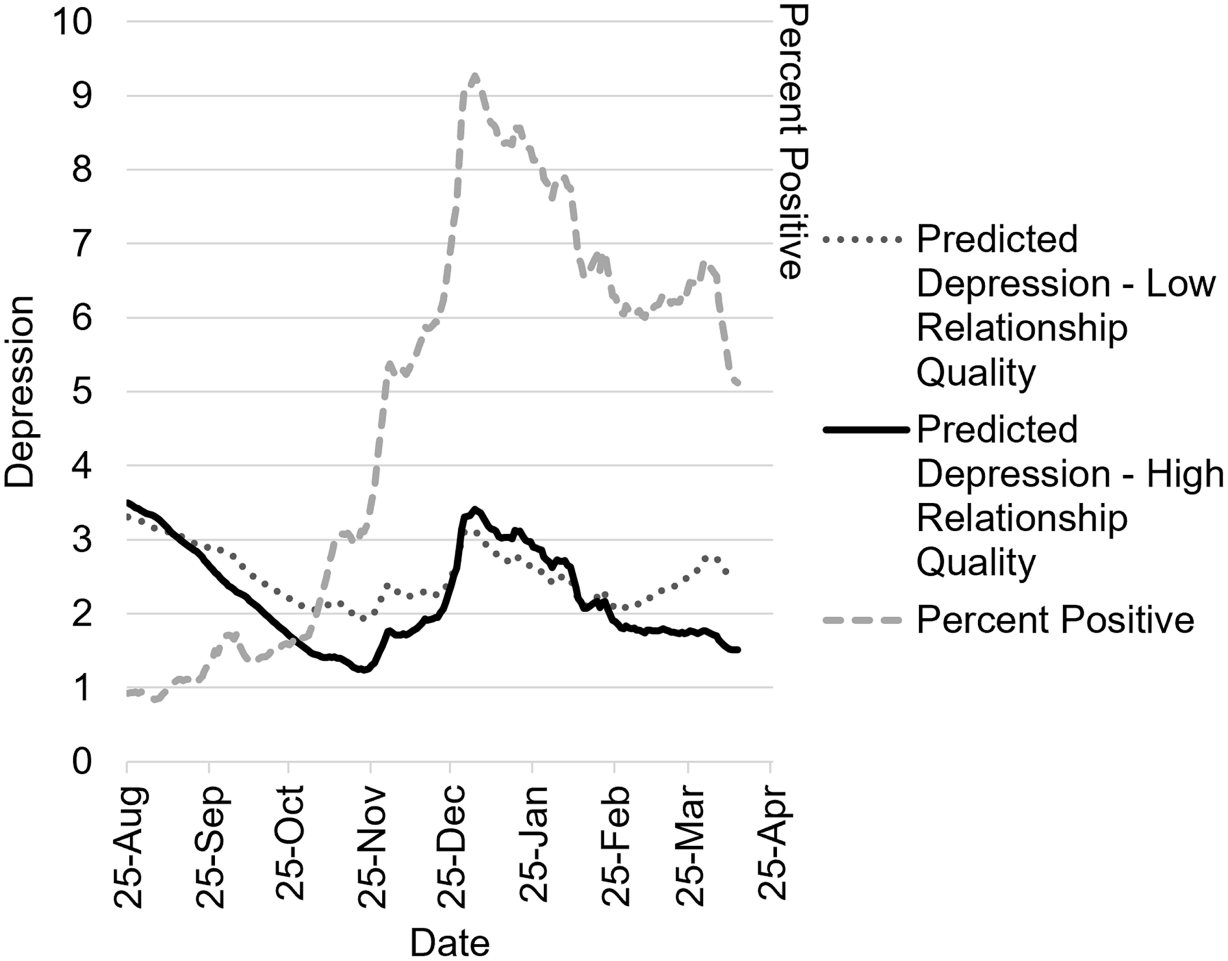
Three-way interaction model predicting daily depression between August 2020–April 2021 as a function of COVID-19 percent positivity testing rate, season, and relationship quality. Predicted daily depression in individuals with low-quality (−1 *SD*) and high-quality (45; maximum score, since +1 *SD* was slightly outside the range of the scale) relationships as a function of daily percent positive testing rate in NYC and season. Depression scores can range from 1 to 9, and percent positive can range from 0 to 100, but the range of percent positive was 0.84 to 9.14 during the observation period. The jagged shape of the graph comes from modeling the effect of actual reported citywide seven-day percent positivity rate.

Full model results for fixed effects and random effects are in Table 2. In the main effects model, on days with a higher seven-day rolling average citywide COVID-19 positivity rate, individuals also reported greater daily depression, *B* = 0.30, 95% CI 0.13, 0.46, *p* < 0.001, and higher relationship quality was associated with lower depression, *B* = −0.04, 95% CI −0.06, −0.01, *p* = 0.003. Sine and cosine were significant at *p* < 0.001, suggesting temporal variation in daily depression. In the two-way interaction model, sine and cosine interacted significantly with citywide positivity rate, *p* = 0.011, and, *p* = 0.048, respectively. The final model included a significant three-way interaction of sine, relationship quality, and citywide positivity rate, *p* = 0.010, and a marginally significant three-way interaction of cosine, relationship quality, and citywide positivity rate, *p* = 0.083; this is depicted in Figure 4 to aid interpretation. Daily depression is elevated during August 2020, but falls steadily over subsequent months, through November, with those in high-quality relationships exhibiting a steeper decline. When cases spike in December, depression also spikes for all couples. As COVID positivity declines again in January – March, depression also decreases, and this is more apparent for individuals in high-quality relationships.

**Table 2:** Estimates for main effects model (column 1), two-way interactions (column 2), and three-way interactions (column 3) predicting daily depression.

	*B* [95% CI]	*B* [95% CI]	*B* [95% CI]
*Intercept*	2.83 [1.60, 4.06]**	0.45 [1.38, 7.71]**	1.50 [−2.52, 5.52]
*Weekend*	−0.18 [−0.29, −0.08]**	−0.18 [−0.28, −0.07]**	−0.18 [−0.28, −0.07]**
*COVID Positive*	0.13 [−0.21, 0.47]	0.19 [−0.15, 0.53]	0.19 [−0.15, 0.53]
*Sine*	0.93 [0.45, 1.41]**	2.55 [−0.37, 5.47]+	−1.38 [−6.01, 3.24]
*Cosine*	−0.13 [−1.64, −0.62]**	−0.23 [−2.88, 2.41]	0.23 [−3.41, 3.86]
*Relationship Quality (RQ)*	−0.04 [−0.06, −0.01]**	−0.07 [−0.15, 0.01]+	0.00 [−0.09 0.10]
*Percent Positivity Rate (PPR)*	−0.30 [0.13, 0.46]**	−0.19 [−1.01, 0.63]	1.12 [−0.11, 2.34]+
RQ*PPR		0.01 [−0.01, 0.03]	−0.02 [−0.05, 0.01]
Sine*RQ		−0.02 [−0.09, 0.05]	0.08 [−0.04, 0.19]
Cosine*RQ		−0.03 [−0.09, 0.04]	−0.04 [−0.13, 0.05]
Sine*PPR		−0.24 [−0.43, −0.05]*	1.03 [0.06, 1.99]*
Cosine*PPR		0.16 [0.00, 0.32]*	−0.71 [−1.70, 0.29]
Sine*RQ*PPR			−0.03 [−0.05, −0.01]**
Cosine*RQ*PPR			0.02 [−0.00, 0.05]+
	*Est (se)*	*Est (se)*	*Est (se)*
*Intercept Variance (Common to Each Dyad Member)*	3.59 (0.60)**	4.11 (0.71)**	4.05 (0.70)**
*Between Dyad Member Intercept Covariance*	1.41 (0.60)*	1.91 (0.71)**	1.85 (0.70)**
*Slope Variance (Common to Each Dyad Member)*	0.19 (0.05)**	0.22 (0.06)**	0.22 (0.06)**
*Between Dyad Member Slope Covariance*	0.09 (0.05)*	0.12 (0.06)*	0.12 (0.06)*
*Within Dyad Member Intercept-Slope Covariance*	−0.74 (0.18)**	−0.88 (0.22)**	−0.88 (0.22)**
*Between Dyad Member Intercept-Slope Covariance*	−0.35 (0.17)*	−0.47 (0.21)*	−0.47 (0.21)*
*Daily Residual Variance (Common to Each Dyad Member)*	1.07 (0.05)**	1.07 (0.05)**	1.07 (0.05)**
*Between Dyad Member Daily Residual Covariance*	0.34 (0.05)**	0.32 (0.05)**	0.32 (0.05)**

The top panel contains fixed effects, and the bottom panel contains random effects.

+*p* < 0.10, *p < 0.05, **p < 0.01.

We next estimated the association for the simple main effect of relationship quality with daily depression for the three-way interaction model on the 5^th^ of each month. Full results for these tests are in Supplementary Table 1. On October 5^th^, relationship was associated with marginally lower daily depression, *p* = 0.052. This association was negative and statistically significant on November 5^th^, *B* = −0.06, 95% CI −0.11, −0.00, *p* = 0.034, and on April 5^th^, *B* = −0.10, 95% CI −0.15, −0.04, *p* < 0.001. Stated otherwise, couples with higher (v. lower) relationship quality experienced decreased depression on the 5^th^ of October, November, and April.

### Sensitivity analyses

Sensitivity analyses modeling time as a count variable (0, 1, 2, etc.) rather than sine and cosine of the date, including both the linear and squared term, replicated the two-way interactions for anxiety and the three-way interactions for depression (see Supplementary Figures 1, 2). Conclusions from these two-and three-way interactions of continuous time with relationship quality and infection rates remained unaltered when additionally controlling for season as a categorical variable.

Only 15 individuals were fully vaccinated (6.5% of sample) and 10 individuals were partially vaccinated (4.3% of sample). Conclusions for the results of the study were unaltered when adjusting for vaccination status. Conclusions were unaltered in models including demographic covariates.

## Discussion

In a sample of cohabiting couples living in NYC during the COVID-19 pandemic, results of this analysis support the idea that greater environmental exposure to COVID-19 stressors, defined as daily percent positivity rate, is associated with increases in daily reports of anxiety and depression. Informed by Ecological Systems Theory (Bronfenbrenner, 1992), this work situates individual well-being within an environmental and relational context (i.e., an individual’s exosystem and mesosystem), expanding our knowledge of how these interacting contexts shape anxiety and depression. Results showed that even small changes in daily COVID-19 positivity rates relate to daily fluctuations in negative affect. However, patterns also indicate some evidence for habituation because mental health outcomes were worst in August of 2020, despite the greatest positivity rates in this dataset occurring in the winter months of 2020–2021. Counter to predictions, there was no evidence for improvement in mental health outcomes as seasons became warmer, despite the ostensible ability for individuals to engage in outdoor activities and escape the confines of their homes. High-quality relationships appeared to offer some protection against elevations in anxiety and depression, particularly when cases were low; that is, when cases were low, couples who reported higher relationship quality showed the lowest rates of daily anxiety and depression. However, there was evidence of *greater* anxiety for couples in high-quality relationships when COVID-19 cases spiked, such that couples with high-quality relationships may have been the most sensitive to changes in citywide COVID-19 cases – at least in terms of daily anxiety.

Exposure to the environmental stressor of the COVID-19 pandemic has varied over time, with rising and falling cases (Hong et al., 2021; COVID-19, 2022), and anxiety and depression seem to mirror these positivity levels – at least within couples cohabiting in NYC. Habituation effects were present, which is aligned with some prior work (Sauer et al., 2020) and expands this to the domain of COVID-19 as a stressor. Despite the greatest case load occurring in the winter months, daily reports of anxiety and depression did not appreciably exceed those levels reported at the beginning of the study. We were surprised to see that mental health was not better during warmer months. It is possible that we were unable to capture these effects since data collection began during August of 2020, the first summer of the pandemic, and we did not capture data in the warmest summer months of 2021 (data collection stopped in April 2021). Despite this limitation, the nine-month data collection period spanned considerable seasonal variation in temperature. It may be that anxiety and depression were declining leading up to August, or that anxiety and depression would have remained low in summer of 2021 despite increased positivity rates. It may also be that differences in policies and tactics for managing the pandemic (e.g., the appearance of personal, heated outdoor dining spaces on the streets of NYC) played a key role in preventing additional adverse mental health consequences. For example, indoor dining opened at 25% capacity in September 2020 in NYC. It is also important to note that vaccines became available for the first time in February 2021, and were available to all NYC residents age 30 and over by the end of March (though finding an appointment was not easy, and data collection ended soon after). If data are available, harmonizing a wider range of mental health data from cities across the US (e.g., warmer cities and fewer mandates, such as Houston, TX) over a longer period of time and matching this to data on temperature and local policy would be a fruitful area for future research.

In line with previous research on the protective effects of relationships that are high in quality for well-being (Lillie et al., 2021; Randall et al., 2021) and theories that highlight the stress-buffering potential of supportive relationships (Pearlin et al., 1981; Cohen and Wills, 1985; Taylor, 2011), couples who reported greater relationship quality at baseline were less anxious and depressed than those who were in lower quality relationships over the course of the 14-day observation period. This was most apparent when COVID-19 case positivity rates were lower or declining. When cases were high, couples exhibited similar levels of daily depression regardless of relationship quality. There was also some evidence that high-quality relationships may have provided a context that increased anxiety during the 2020–2021 winter surge in NYC. It is clear, here, that environmental and relational systems interact to impact individual outcomes. Although these findings may seem surprising, these results are aligned with theories on co-rumination, i.e., excessive and repetitive discussion of problems and negative emotions, whereby two individuals talk about issues in a perseverative manner. Co-rumination is simultaneously associated with positive relational outcomes and anxiety and depression (Waller and Rose, 2010; Ames-Sikora et al., 2017; Felton et al., 2019), and couples higher in relationship quality are also more likely to co-ruminate (Felton et al., 2019). It is therefore possible that, in the current dataset, couples high in relationship quality at baseline were more likely to talk about problems, worries, and stressors than those in lower quality relationships, which could increase focus on COVID-related anxieties. Indeed, although co-ruminative discussions can lead to maladaptive emotional dynamics (Rose et al., 2017; DiGiovanni et al., 2021), research with romantic couples indicates that high levels co-rumination are perceived to be supportive (Ames-Sikora et al., 2017).

It may seem unusual that an environmental exposure that individuals may (or may not) have been aware of – seven-day rolling average citywide COVID-19 percent positivity rate – was associated with anxiety and depression, whereas a confirmed or suspected COVID-19 positive case within the couple was not. However, having COVID oneself does not accurately reflect the general social climate (e.g., a person can have COVID-19 but businesses and restaurants are still open with outdoor service), which could better represent local COVID-19 stress and population fears. There is also considerable research showing that anticipating something bad (e.g., fear of exposure to, and infection from, COVID-19) can cause greater anxiety than receiving the bad news itself (e.g., having a diagnosis of COVID-19; Sweeny and Cavanaugh, 2012; Sweeny and Falkenstein, 2015). Power to detect an effect may also have been limited, as only 10.3 percent of couples reported a suspected or confirmed COVID case on any day, and this represented only 4.7 percent of all observations.

### Strengths and limitations

Study strengths include 14-days of dyadic diary data captured in NYC – an early epicenter of the COVID-19 pandemic. Although prior literature has examined mental health in the context of the pandemic (Choi et al., 2020; Ettman et al., 2020; McGinty et al., 2020; Salari et al., 2020; Twenge and Joiner, 2020; Kwong et al., 2021; Nochaiwong et al., 2021; Santabárbara et al., 2021), most work has not tracked the relationship of fluctuations in COVID-19 cases with daily reports of anxiety and depression. Moreover, much of the existing research on relationships and COVID-19 has examined how COVID-19 stressors are destructive for relationship quality (Balzarini et al., 2020; Williamson, 2020; Neff et al., 2021); in the current study, we sought to examine how relationship quality may either buffer or amplify negative affect resulting from exposure to the environmental stressor of COVID-19 positivity rates. Limitations include lack of generalizability to individuals who are not in a cohabiting romantic relationship or who live outside of NYC, or to those who have other people in the home, such as children or roommates. Additional stressors such as school closures or opportunities for socialization with roommates or other family members could have changed the way COVID-19 impacted mental health and well-being. Furthermore, it is not clear if participants were aware of changing positivity rates. That said, we conceptualized this construct as an environmental exposure, or exosystem (Bronfenbrenner, 1992), akin to census-level socioeconomic status or living in a heat island, and not as an individual difference variable. This operationalization provides novel insights into the ways in which couple dynamics shape reactions to environmental stressors, a critical area for future research in a rapidly warming world that will greatly increase exposure to temperature extremes, disease, climate disasters, and more (Caminade et al., 2019; Schiermeier, 2019; Williams et al., 2019; Strauss et al., 2021).

A little over 50% of our sample identified as White, so the current results may also differ depending on race, ethnicity, or cultural values that impact how individuals express negative emotions (Butler et al., 2007; Matsumoto et al., 2008; Shiota et al., 2010). Future research may look to specifically study individuals from more marginalized backgrounds, as the current study surveyed a convenience sample. Often, couples who are more satisfied with their relationship are more likely to participate in research together, which could have contributed to range restriction and limited the inclusion of couples in lower quality relationships.

Data were also limited in that they captured only August 2020 through April 2021. Capturing more data over a longer period of time may have uncovered additional trends based on seasonality or other habituation effects as vaccines became widely available. Couples also contributed only two weeks of data each; thus, it is possible that couples changed in systematic ways over time (i.e., those who joined the study later may be different than those who joined earlier; however, supplemental analyses [not shown] uncovered no significant association of start date for the daily diary with age or relationship quality). Still, the present study provides some of the first data examining individual well-being within interaction environmental and relational systems. Future research on the ways in which global environmental stressors shape well-being over time should consider capturing repeated-measures data over a longer period. Finally, because data were captured daily, to reduce participant burden, we did not include clinical assessments of anxiety and depression.

## Conclusion

COVID-19 caused unprecedented disruption to daily life for many, especially those living in NYC – an early epicenter of the COVID-19 pandemic. The present study tested the association of citywide COVID-19 positivity rate with 14-day daily diary reports of anxiety and depression in romantic couples cohabiting in NYC during the months of August 2020 through April 2021. Results showed that increases in anxiety and depression mirrored spikes in COVID-19 positivity rate. Given the acute, negative impact of a novel environmental stressor on well-being, our findings emphasize the need for intervention early during exposure to these profoundly disruptive global events. Romantic relationships may be a central context for bolstering individuals support systems and promoting resilience during times of social isolation (Pearlin et al., 1981; Cohen and Wills, 1985; Taylor, 2011), however, our findings also indicate that high quality relationships could incidentally increase negative affect under some environmental circumstances (i.e., when COVID-19 rates were high). Care must be taken to promote supportive relationships where couples can discuss stressors without catalyzing cycles of co-rumination that can exacerbate anxiety.

## Data availability statement

The datasets presented in this study can be found in online repositories. The names of the repository/repositories and accession number(s) can be found at: https://osf.io/uyfmk/?view_only=2214b2e5522a4e9caf3cf654cb420dff.

## Ethics statement

The studies involving human participants were reviewed and approved by Columbia University. The ethics committee waived the requirement of written informed consent for participation.

## Author contributions

TC: conceptualization, formal analysis, investigation, methodology, visualization, writing—original draft, and writing—review and editing. AD: data curation, investigation, methodology, and writing—review and editing. NB: methodology, supervision, and writing—review and editing. All authors contributed to the article and approved the submitted version.

## Funding

This work was supported by the National Institutes of Health Science of Behavior Change Program through an award administered by the National Institute on Aging (U24AG052175). Cornelius receives support from the National Institutes of Health National Center for Advancing Translational Sciences (KL2 TR001874). The content is solely the responsibility of the authors and does not necessarily represent the official view of the NIH.

## Conflict of interest

The authors declare that the research was conducted in the absence of any commercial or financial relationships that could be construed as a potential conflict of interest.

## Publisher’s note

All claims expressed in this article are solely those of the authors and do not necessarily represent those of their affiliated organizations, or those of the publisher, the editors and the reviewers. Any product that may be evaluated in this article, or claim that may be made by its manufacturer, is not guaranteed or endorsed by the publisher.

## Footnotes

^1^ https://osf.io/uyfmk/?view_only=2214b2e5522a4e9caf3cf654cb420dff

^2^ https://osf.io/uyfmk/?view_only=2214b2e5522a4e9caf3cf654cb420dff
