# Supplementary material for: The associations of COVID-19 percent positivity rate, relationship quality, and season with daily anxiety and depression in couples living in NYC
Source: Front Psychol. 2022 Dec 22;13:968243. doi: 10.3389/fpsyg.2022.968243 (PMC9813508; doi:10.3389/fpsyg.2022.968243)
Supplement: Supplementary file 1 [file Data_Sheet_1.DOCX]

**Supplemental Table 1.** Simple main effect of relationship quality on the 5^th^ of each month for two- and three-way interaction models predicting daily anxiety and the three-way interaction model predicting daily depression.

+*p* < .10, **p* < .05, ***p* < .01

|  | *September 5, 2020* | *October 5, 2020* | *November 5, 2020* | *December 5, 2020* | *January 5, 2021* | *February 5, 2021* | *March 5, 2021* | *April 5, 2021* |
| --- | --- | --- | --- | --- | --- | --- | --- | --- |
|  | *B* [95% CI] | *B* [95% CI] | *B* [95% CI] | *B* [95% CI] | *B* [95% CI] | *B* [95% CI] | *B* [95% CI] | *B* [95% CI] |
| *2-Way Model: Anxiety* | -0.03  [‑0.09, 0.03] | -0.01  [‑0.06, 0.03] | -0.03  [‑0.08, 0.02] | 0.01  [‑0.05, 0.06] | 0.06  [‑0.04, 0.15] | 0.00  [‑0.06, 0.06] | -0.05 [‑0.10, ‑0.01]* | -0.05  [‑0.10, 0.01] |
| *3-Way Model: Anxiety* | 0.01  [-0.06, 0.08] | -0.03  [-0.07, 0.02] | -0.05  [-0.11, 0.01]+ | 0.00  [-0.08, 0.08] | 0.10  [-0.01, 0.21]+ | 0.03  [-0.04, 0.11] | -0.04  [-0.09, 0.01]+ | -0.08  [‑0.15, ‑0.01]* |
| *2-Way Model: Depression* | 0.01  [-0.05, 0.08] | -0.04  [‑0.09, 0.00]+ | -0.06  [‑0.11, ‑0.00]* | -0.05  [‑0.12, 0.02] | 0.03  [‑0.05, 0.11] | 0.02  [‑0.03, 0.07] | -0.03  [‑0.07, 0.01] | -0.10  [‑0.15, ‑0.04]** |

**Supplemental Figure 1.** Two-way interaction model predicting daily anxiety between August 2020 – April 2021 as a function of COVID-19 percent positivity testing rate, continuous time, and relationship quality.


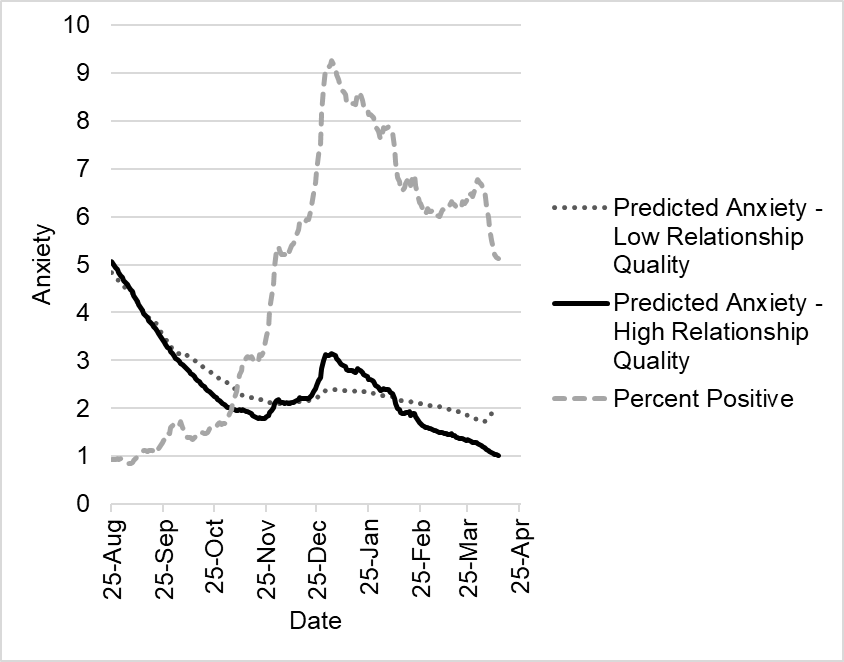


Percent Positive

*Note.* Predicted daily anxiety in individuals with low-quality (-1 *SD*) and high-quality (45; maximum score, since +1 *SD* was slightly outside the range of the scale) relationships as a function of daily percent positive testing rate in NYC and continuous time. Anxiety scores can range from 1 to 9, and percent positive can range from 0 to 100, but the range of percent positive was 0.84 to 9.14 during the observation period. The jagged shape of the graph comes from modeling the effect of actual reported citywide seven-day percent positivity rate.

**Supplemental Figure 2.** Three-way interaction model predicting daily depression between August 2020 – April 2021 as a function of COVID-19 percent positivity testing rate, continuous time, and relationship quality.


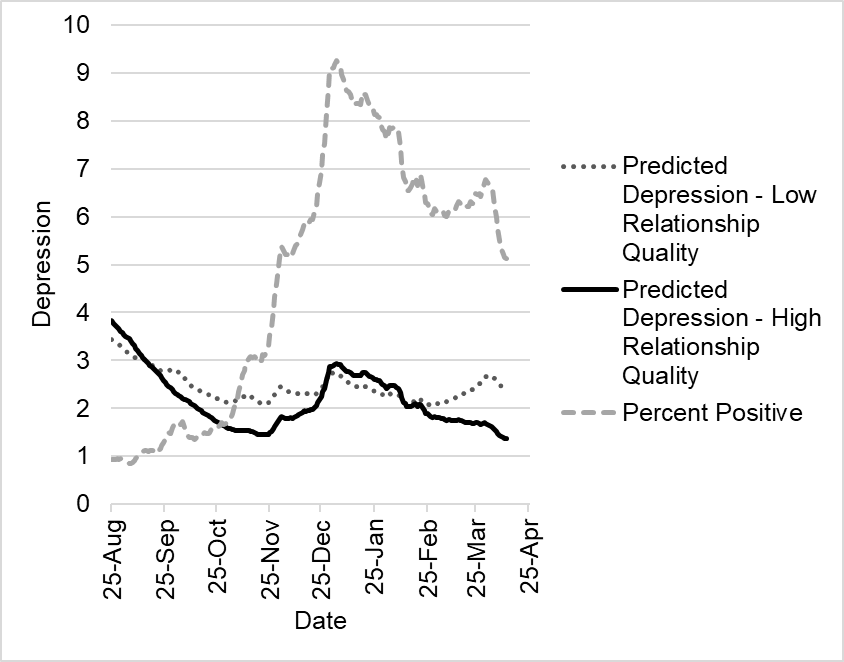


Percent Positive

*Note.* Predicted daily depression in individuals with low-quality (-1 *SD*) and high-quality (45; maximum score, since +1 *SD* was slightly outside the range of the scale) relationships as a function of daily percent positive testing rate in NYC and continuous time. Depression scores can range from 1 to 9, and percent positive can range from 0 to 100, but the range of percent positive was 0.84 to 9.14 during the observation period. The jagged shape of the graph comes from modeling the effect of actual reported citywide seven-day percent positivity rate.
